# The regulatory subunits of CK2 complex mediate DNA damage response and virulence in *Candida Glabrata*

**DOI:** 10.1186/s12866-023-03069-4

**Published:** 2023-10-28

**Authors:** Qi Ni, Xianwei Wu, Tongxuan Su, Cen Jiang, Danfeng Dong, Daosheng Wang, Wei Chen, Yingchao Cui, Yibing Peng

**Affiliations:** 1 Department of Laboratory Medicine, Ruijin Hospital, Shanghai Jiaotong University School of Medicine, No.197 Ruijin ER Road, Shanghai, 200025 China; 2 Institute of Respiratory Diseases, Longhua Hospital, Shanghai University of Traditional Chinese Medicine, No.725 South Wanping Road, Shanghai, 200032 China

**Keywords:** *Candida glabrata*, DNA damage, Cell cycle, Macrophage, Virulence

## Abstract

**Background:**

*Candida glabrata* which belongs to normal microbiota, has caused significant concern worldwide due to its high prevalence and drug resistance in recent years. *C. glabrata* has developed many strategies to evade the clearance of the host immune system, thereby causing persistent infection. Although coping with the induced DNA damage is widely acknowledged to be important, the underlying mechanisms remain unclear.

**Results:**

The present study provides hitherto undocumented evidence of the importance of the regulatory subunits of CgCK2 (CgCkb1 and CgCkb2) in response to DNA damage. Deletion of Cg*CKB1* or Cg*CKB2* enhanced cellular apoptosis and DNA breaks and led to cell cycle delay. In addition, deficiencies in survival upon phagocytosis were observed in Δ*ckb1* and Δ*ckb2* strains. Consistently, disruption of Cg*CKB1* and Cg*CKB2* attenuated the virulence of *C. glabrata* in mouse models of invasive candidiasis. Furthermore, global transcriptional profiling analysis revealed that CgCkb1 and CgCkb2 participate in cell cycle resumption and genomic stability.

**Conclusions:**

Overall, our findings suggest that the response to DNA damage stress is crucial for *C. glabrata* to survive in macrophages, leading to full virulence in vivo. The significance of this work lies in providing a better understanding of pathogenicity in *C. glabrata*-related candidiasis and expanding ideas for clinical therapies.

**Supplementary Information:**

The online version contains supplementary material available at 10.1186/s12866-023-03069-4.

## Background

The incidence of candidiasis infections has increased progressively in recent decades. As the second most frequent pathogenic yeast associated with candidiasis, *Candida glabrata* has several unique biological features, including absence of hyphae, antifungal resistance, and virulence factors. Unlike *Candida albicans*, *C. glabrata* is a haploid budding yeast similar to the non-pathogenic yeast *Saccharomyces cerevisiae* evolutionarily [1]. *C. glabrata* now accounts for one-third or more of all candidemia isolates in USA and a trend of increasing *C. glabrata* rates is seen in Australia and in some European and Asia countries as well, which even causes a mortality rate of 30% [2]. Moreover, reduced susceptibility to azoles and acquired cross-resistance during clinical treatment have been observed in *C. glabrata*. *C. glabrata* has caused significant health concerns worldwide based on increasing evidence of its high prevalence and associated drug resistance [3, 4].

As part of the normal flora, *C. glabrata* often causes superficial skin and mucosal infection and even leads to life-threatening invasive infections, especially in immunocompromised patients. *C. glabrata* is an opportunistic pathogen with secretory hydrolytic and proteolytic enzymes and adhesion and biofilm formation abilities [5, 6]. In contrast with *C. albicans, C. glabrata* lacks many virulence factors but is highly evolved for interaction with the host. *C. glabrata* is a pathogen that induces a moderate immune response to prolong the disease course [7, 8]. Both innate immune and adaptive immune responses contribute to the host defenses of *C. glabrata.* Encountering phagocytic cells and further survival within them is crucial for keeping *C. glabrata* shielded from immune attack [9]. After being phagocytized, *C. glabrata* is confronted with several challenges. DNA damage under a series of phagocytic stress factors (such as reactive oxygen species and cytokines) has been documented within 2 h of phagocytosis. *C. glabrata* induces chromatin remodeling to manage the stress of DNA damage and even replicates inside macrophages after adapting to the hostile environment to cause stubborn infections [10]. Responses to DNA damage including cell cycle checkpoints and DNA repair pathways are crucial for maintaining genetic integrity and are highly diverse in fungi [11].

CK2 is a conserved ubiquitous serine/threonine kinase protein complex and is a heterotetramer composed of two catalytic subunits (Cka1 and Cka2, α and α’ subunit) which bind to the dimer of regulatory subunits (Ckb1 and Ckb2, β subunits) [12]. CK2 participates in various activities such as cell cycle regulation, cell stress response, and apoptosis [13]. In addition to the holoenzyme, the functions of individual subunits have been confirmed [14]. It has been reported that CK2 was recognized as a key director in DNA repair process of both single-strand break (SSB) and double-strand break (DSB) [15, 16]. Moreover, DNA damage repair pathways could be blocked by the inhibitors of CK2 [17]. Many recent studies have found that ScCK2 can directly phosphorylate downstream proteins and is involved in histone phosphorylation [18, 19]. The β subunit of ScCK2 in *S. cerevisiae* has a zinc finger structure, which may be related to the structural stability of the CK2 complex [20, 21]; the C-terminal of β subunits plays an important role in the interaction with catalytic subunits and other kinases, and may also be related to the recruitment of downstream target proteins [13]. The deletion of Sc*CKB1* and Sc*CKB2* results in the cells’ inability to recover or adapt to DSB [12]. In *C. albicans*, CaCka1 and CaCka2 have been observed to govern virulence and azole drug resistance, but the significance of the regulatory subunits of CK2 remains unknown [22]. Overall, the *C. glabrata* CK2 complex has been largely understudied over the past few years, warranting further research.

In the present study, the phenotypes of Cg*CKB1* and Cg*CKB2* null mutants in relation to stress survival and virulence were examined. In addition, global transcriptome profiling was performed by RNA-seq to investigate the molecular regulations in CgCkb1 and CgCkb2. We show for the first time that in *C. glabrata*, the β subunits of CK2 are crucial in DNA damage response possibly by maintaining a normal cell cycle. Moreover, our data suggest a link between genome integrity and survival upon phagocytosis, which is important for full virulence of *C. glabrata in vivo*.

## Results

### *C. glabrata* Ckb1 and Ckb2 are phylogenetically closely related to *S. cerevisiae* Ckb1 and Ckb2

**Fig. 1:**
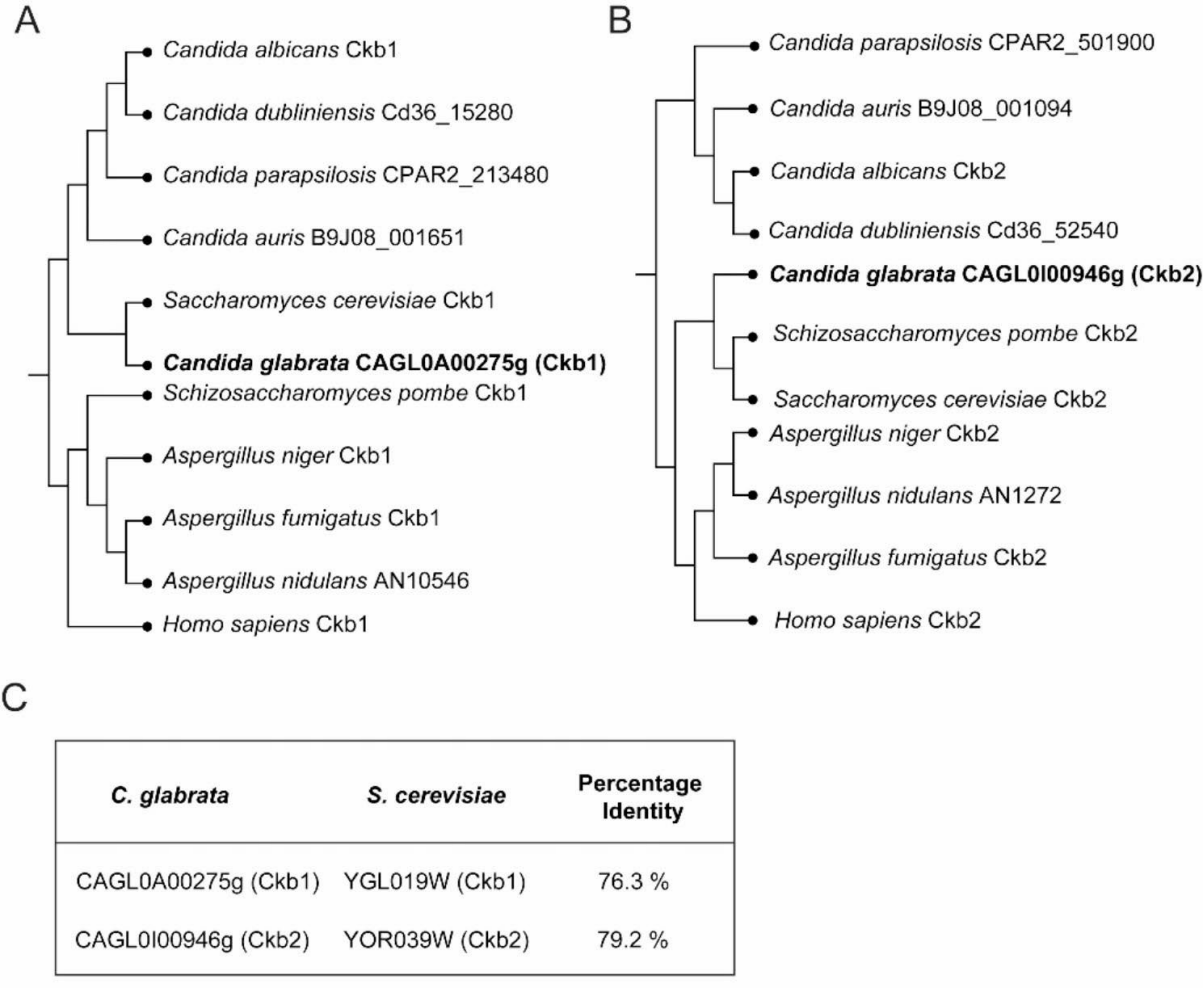
Phylogenetic trees of fungal Ckb1and Ckb2. (**A**) and (**B**) A phylogenetic tree constructed based on fungal Ckb1 or Ckb2 protein sequences to show the sequence homology of Ckb1 or Ckb2 proteins across species. The human Ckb1 or Ckb2 protein sequence was used as the root, and the maximum likelihood method was used for calculation and comparison by MEGA (V11.0.13). (**C**) Overview of amino acid identity between the *C. glabrata* and *S. cerevisiae* Ckb1 and CKb2.

Although being the most common pathogens of candidiasis, *C. albicans* and *C. glabrata* are from different evolutionary clades. *C. albicans* belongs to the CTG clade, which includes pathogens like *C. parapsilosis*, *C. dubliniensis* and *C. auris*. However, *C. glabrata* belongs to the Nakaseomyces clade, which has a higher interspecific affinity for *S. cerevisiae* than *C. albicans*. Therefore, we used the *Saccharomyces cerevisiae* Ckb1/Ckb2 protein sequences to retrieve the Ckb1/Ckb2 protein sequences in BLASTP for various fungi, including *C. glabrata*. Phylogenetic trees of these protein sequences were constructed to show the sequence homology of the Ckb1/Ckb2 proteins across species (Fig. 1). For Ckb1 (CAGL0A00275g) and Ckb2 (CAGL0I00946g) of *C. glabrata*, the *S. cerevisiae* Ckb1 and Ckb2 were identified as closest orthologs respectively. The amino acid sequence of the Ckb1/Ckb2 protein from *C. glabrata* was highly similar to that of *S. cerevisiae* (Ckb1 76.3%, Ckb2 79.2%). The close relationship between *C. glabrata* Ckb1/ Ckb2 and ScCkb1/ScCkb2 suggests that the β subunit of CK2 of *C. glabrata* may have similar phenotypes as in *S. cerevisiae*.

### Cg*CKB1* and Cg*CKB2* genes are essential in response to DNA damage

**Fig. 2:**
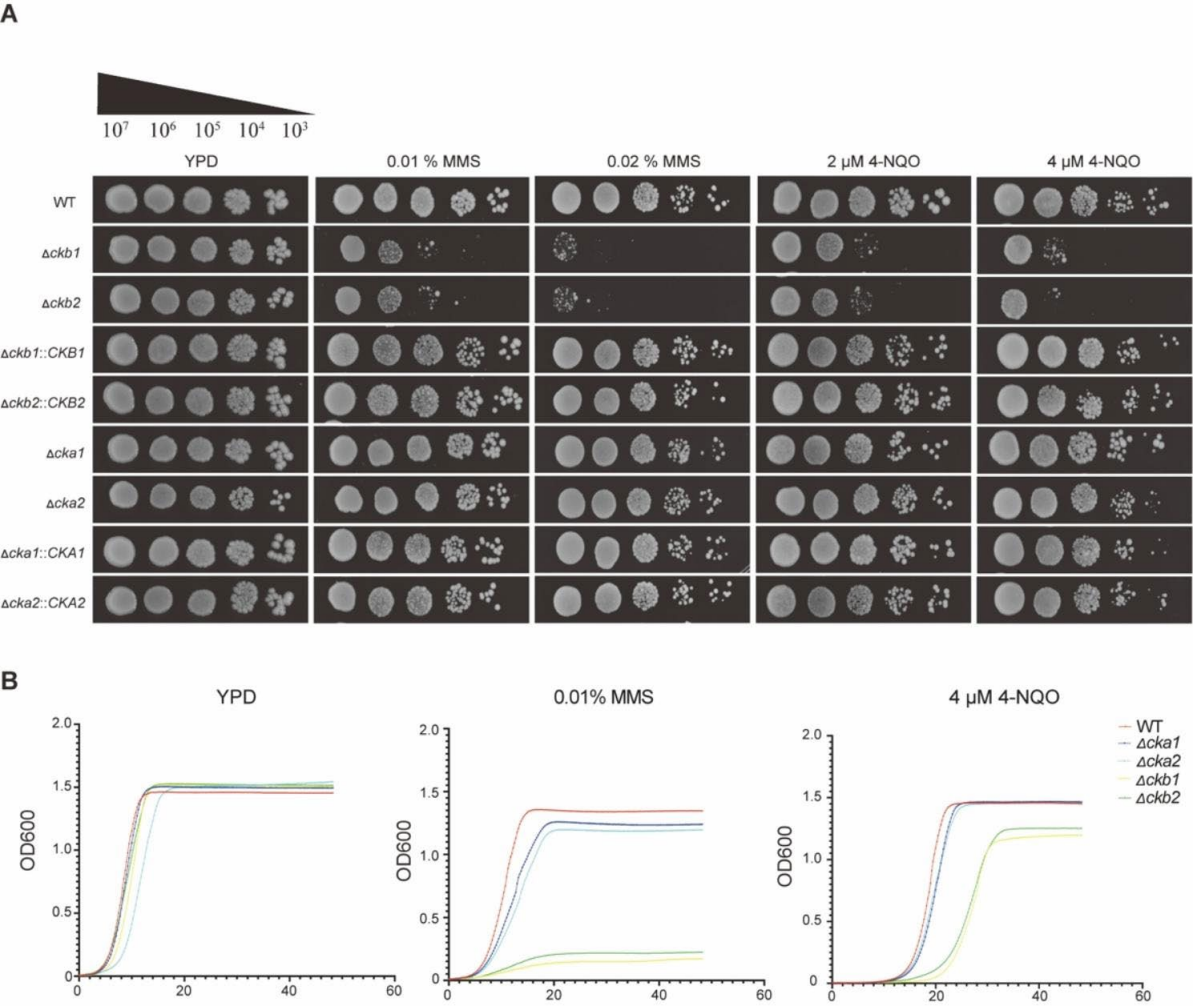
*CgCKB1* and *CgCKB2* genes are essential in response to DNA damage. (**A**) Strains were diluted serially in 10-fold to being grown on YPD containing different concentrations of DNA damage reagents (MMS, methyl methane sulfonate and 4-NQO, 4-Nitroquinoline-1-oxide) at 30℃ for 2 days. WT, wildtype. (**B**) Growth curves of each strain after treatment with different concentrations of DNA damage reagents. The OD600 values were obtained by BioTek plate reader every 15 min at 30 °C for 48 h. Data were representative of three independent experiments. All the growth curves were repeated at least three times.

DNA damage reagents MMS (Methyl Methane Sulfonate) or 4-NQO (4-Nitroquinoline-1-oxide) were supplemented to YPD plates to examine the DNA repair ability of WT, Δ*ckb1*, and Δ*ckb2* strains. The Cg*CKB1* or Cg*CKB2* null mutants exhibited increased susceptibilities to DNA damage reagents than WT strains, and these differences became more obvious as the concentration of reagents increased (Fig. 2A). In contrast, no differences were observed in WT strains lacking Cg*CKA1* or Cg*CKA2* genes encoding catalytic subunits of the CK2 complex (Fig. 2A).

In addition, growth curves of WT, Δ*ckb1*, and Δ*ckb2* strains cultivated with different media revealed similar results with spot assays (Fig. 2B). Under the treatment of 0.01% MMS or 4 µM 4-NQO, the Δ*ckb1* and Δ*ckb2* strains grew slowly and reached significantly lower OD_600_ (Optical density) values in the plateau phase than WT. In contrast, Δ*cka1* and Δ*cka2* exhibited similar growth patterns with WT. Consistent results were observed in RPMI 1640 medium (Figure S1). These results suggest that the regulatory subunits of the CK2 complex might participate in the response to DNA damage.

### Deletion of Cg*CKB1* or Cg*CKB2* increases cellular apoptosis and DNA breaks

**Fig. 3:**
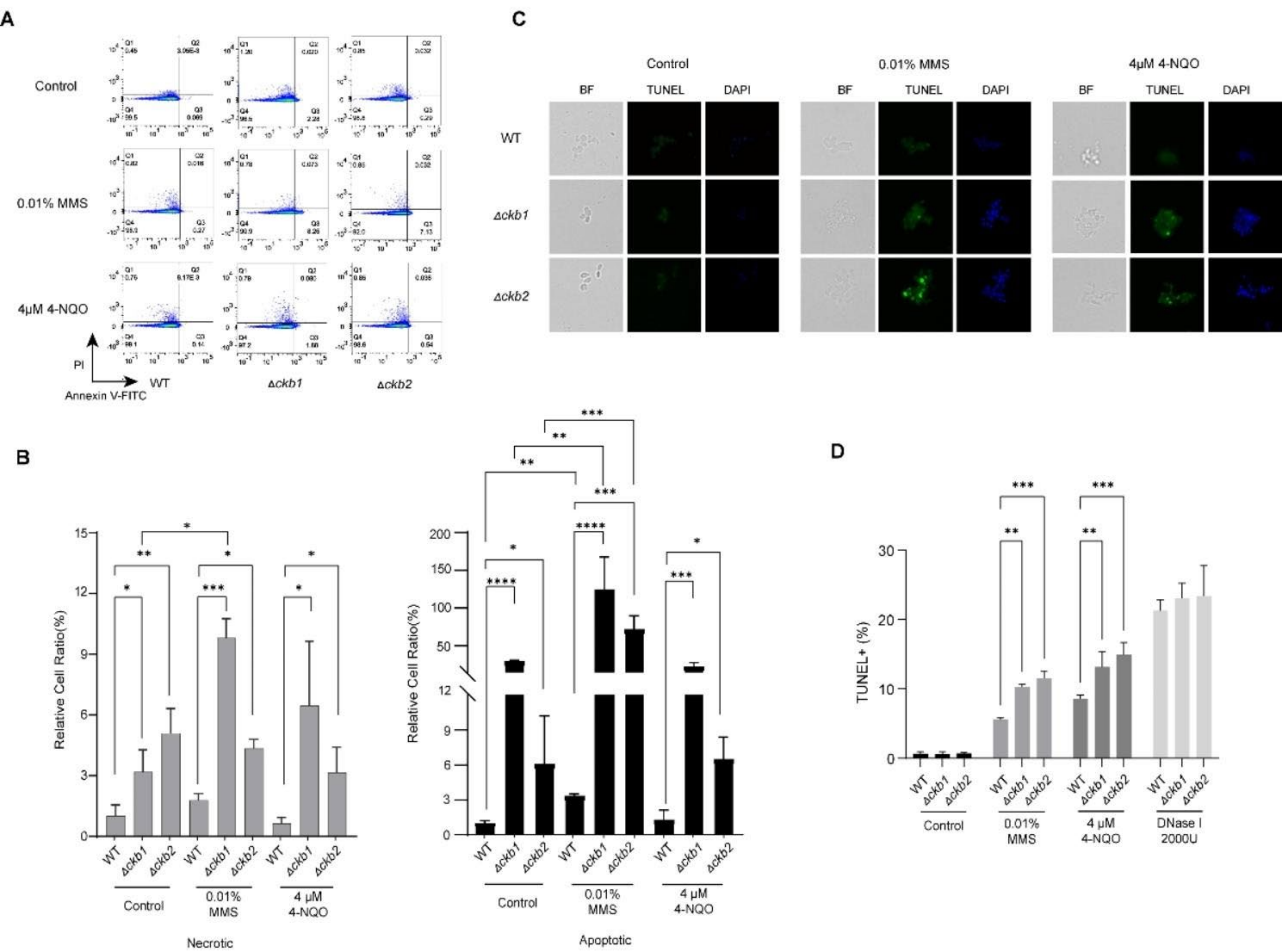
Deletion of *CgCKB1* or *CgCKB2* increases cellular apoptosis and DNA breaks. (**A**) Cells were treated with 0.01% MMS or 4 µM 4-NQO or left untreated for 2 h and stained with Annexin V-FITC and PI. The Fluorescence intensity was detected by BD Fortessa Flow cytometer. Q1 (Annexin V-/PI+): dead cells; Q2 (Annexin V+/PI+): necrotic cells; Q3 (Annexin V+/PI-): apoptotic cells; Q4 (Annexin V-/PI-): live cells. (**B**) The percentages of each strain that are apoptotic (black bars) and death (gray bars) were obtained. The relative cell ratio was compared with WT in RPMI 1640 medium (set at 1.0) (**C**) Representative fluorescence micrographs showing DNA fragmentation and unusual chromosome agglutination. Cells were incubated in RPMI 1640, 0.01% MMS or 4 µM 4-NQO for 12 h at 30 ℃ and then stained with DAPI and FITC. The DNA fragmentation was visualized with FITC (green fluorescence) and unusual chromosome agglutination was visualized with DAPI (blue fluorescence). Data were representative of three independent experiments. (**D**) The TUNEL-positive cells were quantified. DNase I treatment was used as the positive control. The percentages of TUNEL-positive cells were presented as mean ± SD and assessed for statistical analysis by one-way ANOVA followed by Tukey test; * *p* < 0.05, ** *p* < 0.01, *** *p* < 0.001, **** *p* < 0.0001.

Given that DNA damage induces certain changes in genome integrity, we detected the apoptosis and DNA fragmentation of Cg*CKB1* and Cg*CKB2* null mutants in the presence of DNA damage reagents. Annexin V-FITC was used to investigate the phosphatidylserine (PS) molecules exposed outside the cell membrane to identify apoptotic cells. PI (Propidium Iodide) was used to detect necrotic cells. After treatment with 0.01% MMS and 4µM 4-NQO for 2 h, the apoptosis and necrotic cells were detected by flow cytometry in each group. Larger amounts of apoptotic and necrotic cells were observed in Cg*CKB1* and Cg*CKB2* null mutants than in WT when cultured in the RPMI 1640 medium. Consistent results were observed in the presence of DNA damage stress (Fig. 3A and B). This finding suggests that *CgCKB1* and *CgCKB2* null mutants are more prone to apoptosis in response to DNA-damaging agents.

Furthermore, a TUNEL assay was conducted to detect DNA fragmentation by fluorescence microscope and flow cytometry. Cg*CKB1* or Cg*CKB2* null mutant cells displayed a higher abundance of TUNEL-positive nuclei than WT cells upon 0.01% MMS and 4µM 4-NQO treatment (Fig. 3C). Consistently, the proportions of TUNEL-positive cells significantly increased both in Δ*ckb1* and Δ*ckb2* strains when treated with DNA damage reagents (Fig. 3D). Additionally, the degree of chromosome agglutination by fluorescence intensity was observed by DAPI (Diamidino-phenylindole). In the presence of DNA-damaging reagents, blue fluorescence became brighter in *CgCKB1* or *CgCKB2* mutant cells than in WT cells, which indicated more agglutination of chromosomes (Fig. 3C).

### Cell cycle delay occurs in Cg*CKB1* and Cg*CKB2* null mutants

**Fig. 4:**
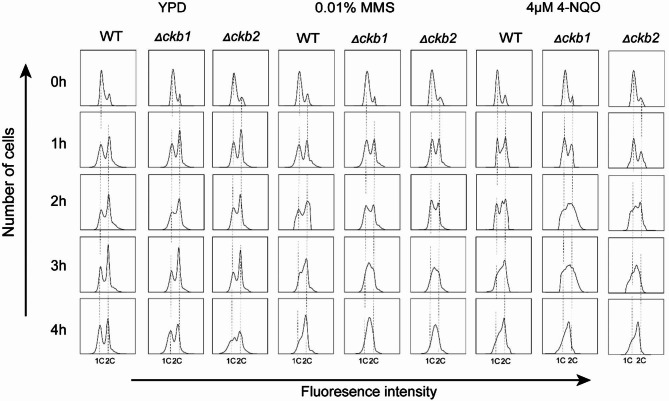
The cell cycle is delayed in Cg*CKB1* and Cg*CKB2* null mutants. Yeast cells were synchronized in YP (carbon starvation condition) and released to YPD either in the absence or presence of 0.01% MMS or 4 µM 4-NQO and collected once an hour. Flow cytometry was used to measure DNA content.

Since deletion of Cg*CKB1* or Cg*CKB2* increases the sensitivity to DNA damage agents, apoptosis and DNA fragmentation in *C. glabrata*, we wondered whether the cell cycle of *CgCKB1* and *CgCKB2* null mutants was altered. A carbon starvation strategy was used to synchronize *C. glabrata* cells at the G1 phase. After being released in the 2% glucose YPD medium with or without 0.01% MMS or 4µM 4-NQO, we used flow cytometry to analyze the distribution of cell cycles once an hour. In the YPD medium, similar cell cycle patterns were observed in WT, Δ*ckb1* and Δ*ckb2* strains. DNA replicated from 1 to 2 C gradually within 3 h. S phase cells slightly accumulated in Δ*ckb1* and Δ*ckb2* at 4 h. In the MMS or 4-NQO-containing YPD medium, both WT and null mutants slowed down DNA replication obviously. In contrast with the YPD culture, a larger proportion of the population remained at the S phase at 2 h under DNA damage stress in both null mutant cells. After 3 h, parts of WT cells completed DNA replication gradually; however, Δ*ckb1* and Δ*ckb2* cells remained at the S phase and failed to replicate DNA within 4 h (Fig. 4 and S2). Taken together, these data demonstrated that regulation of the cell cycle is disrupted in Δ*ckb1* and Δ*ckb2*, and CgCkb1 and CgCkb2 are crucial in maintaining a normal cell cycle upon DNA damage stress.

### Cg*CKB1* and Cg*CKB2* null mutants have defects in replication in macrophages

**Fig. 5:**
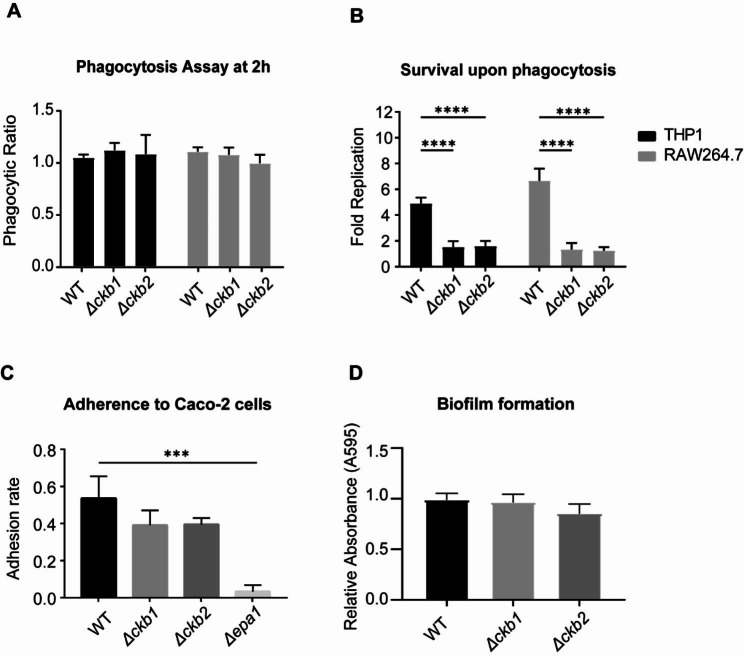
Effect of Cg*CKB1* and Cg*CKB2* on interaction with host cells. (**A**) After co-incubation of fungi with RAW264.7 macrophages or PMA-treated THP-1 cells for 2 h, the cells were lysed with 0.05% Triton X-100 to release the fungi, and the phagocytosis rate was defined as the number of fungi phagocytosed by macrophages after 2 h to the number of fungi before co-incubation. (**B**) After cocultured with macrophages for 24 h, survival upon phagocytosis was measured as described above. The ratio of the intracellular yeast cells after 24 h of coculture to that after 2 h coculture was defined as fold replication. (**C**) Epithelial Caco-2 cells were cocultured with Cg*CKB1* or Cg*CKB2* null mutants, respectively. Δ*epa1* which exhibited deficient adhesion ability was used as a control. (**D**) Log-phased yeast cells were seeded in a 24-well plate for biofilm formation assay. Crystal violet [0.4% (w/v)] was used for staining mature biofilms. The destaining solution was measured for absorbance at 595 nm. Wells without yeast cells were used as background. CBS138 was the used as the WT and the knockout strains were generated using CBS138 as the parent. Experiments were performed three times. Error bars show standard deviation. Comparisons were performed using one-way ANOVA followed by Tukey test, and asterisks indicate statistically significant differences (*** *p* < 0.001, **** *p* < 0.0001).

Dysregulation of Cg*CKB1* or Cg*CKB2* genes leads to defects in response to DNA damage and cell cycle delay in *C. glabrata*. We assessed cell survival upon phagocytosis to further explore whether Cg*CKB1* and Cg*CKB2* play essential roles in interacting with the host immune system. PMA (Phorbol 12-myristate 13-acetate) -induced human monocytic cell line THP-1 and mouse leukemia macrophage cell line RAW 264.7 were used in this study. Similar numbers of WT, Δ*ckb1* and Δ*ckb2* cells were engulfed after co-incubated with macrophages for 2 h. Nevertheless, WT cells yielded 4.90 -fold and 6.66-fold higher replication rates after cocultured with THP-1 and RAW 264.7 cells for 24 h, respectively (Fig. 5A). In contrast, Cg*CKB1* or Cg*CKB2* null mutants exhibited only a 1.53- or 1.60-fold increase in replication in THP-1 cells (*p* < 0.0001) and 1.34-fold or 1.25-fold (*p* < 0.0001) increase in RAW264.7 cells (Fig. 5B). These results substantiated that Δ*ckb1* and Δ*ckb2* strains induced dysregulated macrophage replication, and Cg*CKB1* and Cg*CKB2* are required for intracellular survival in macrophage.

In addition, we evaluated the adhesion ability to epithelial cells Caco-2 and biofilm formation in WT, Δ*ckb1*, and Δ*ckb2* strains. The adhesion rates of Δ*ckb1* and Δ*ckb2* strains were slightly reduced, although the difference was not statistically significant (*p* > 0.05) (Fig. 5C). Moreover, Δ*ckb1* and Δ*ckb2* strains displayed similar biofilm formation abilities to WT (Fig. 5D).

### Cg*CKB1* and Cg*CKB2* are important in the virulence of systemic candidiasis mouse model

**Fig. 6:**
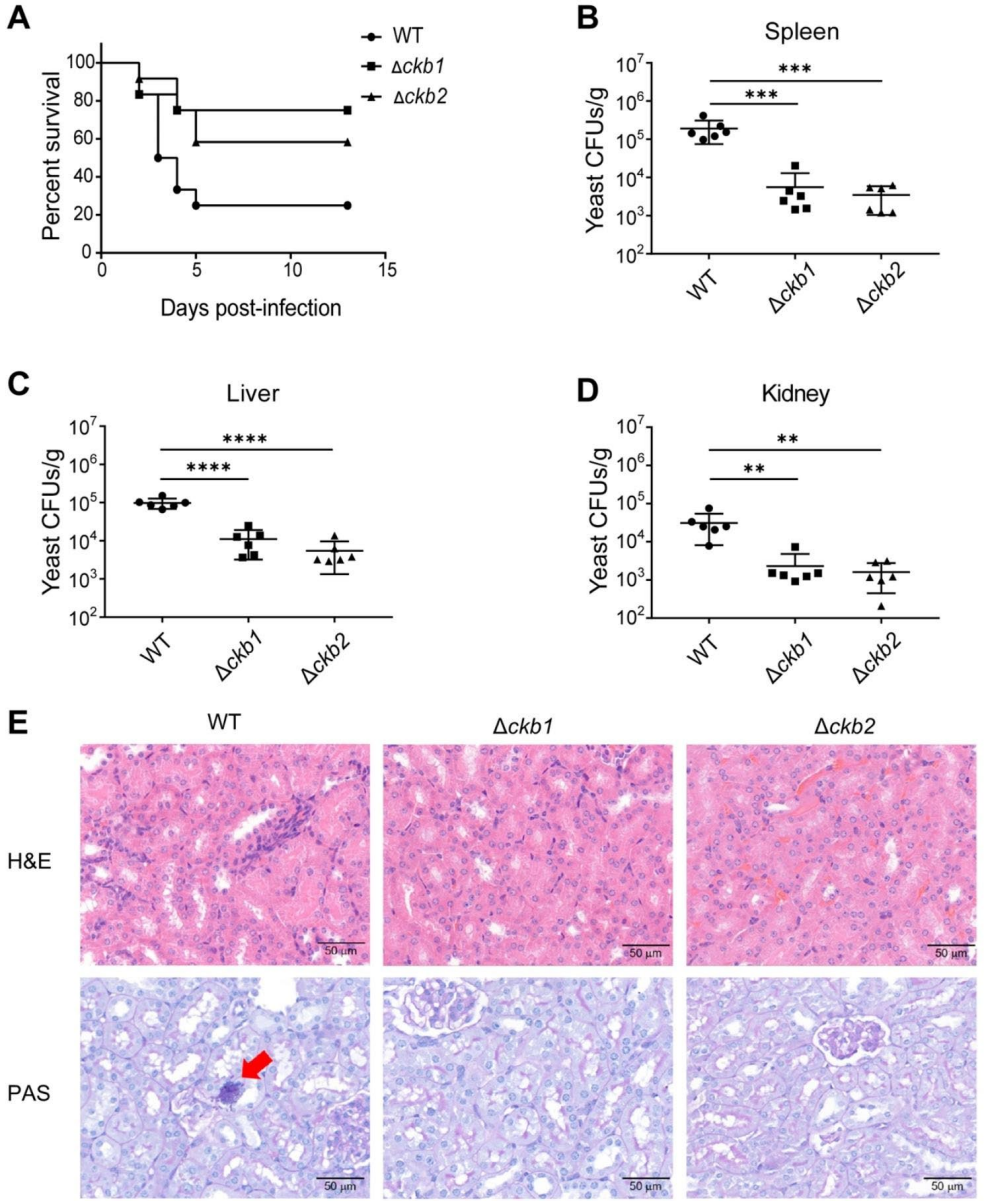
*CgCKB1* and *CgCKB2* are essential to virulence in vivo. (**A**) 200 mg/kg cyclophosphamide was intraperitoneally injected per ICR mouse (5–6 weeks old, 24–26 g) on day − 3 and every fourth day later. Then mice were infected with 1 × 10^8^ WT, Δ*ckb1* or Δ*ckb2* cells in 200 µL in 0.9% (w/v) saline (n = 12). Mice in the agonal stage were humanely euthanized by cervical dislocation. Experiments were terminated on day 14. Analysis by the Gehan-Breslow-Wilcoxon test indicated that in contrast to WT, the virulence of Cg*CKB1* and Cg*CKB2* null mutants was significantly attenuated (*p* < 0.05). (**B**)(**C**)(**D**) Fungal burden assays were performed using ICR mice (n = 6). ICR mice were immunosuppressed with 200 mg/kg cyclophosphamide on day − 3 and were further injected with 5 × 10^7^ yeast cells through the tail veil on day 0. Organs were harvested, weighed and mechanically homogenized 3 days post-infection. Tissue homogenates were diluted, plated onto YPD agar, and incubated at 30℃ for 2 days. CFUs were calculated and analyzed. Error bars show standard deviation. Comparisons were performed using one-way ANOVA followed by Tukey test, and asterisks indicate statistically significant differences (**: *p* < 0.01; ***: *p* < 0.001, ****: *p* < 0.0001). CBS138 was the used as the WT and the knockout strains were generated using CBS138 as the parent; (**E**) Representative HE (Hematoxylin-Eosin) and PAS (Periodic Acid-Schiff) stained sections of kidneys from immunosuppressed ICR mice on day 3 post-infection. Magnification was ×20. PAS-positive yeast cells were indicated by red arrowhead. Data are representative of at least three replicates.

We used a systemic candidiasis mouse model through tail vein injection to investigate the importance of Cg*CKB1* and Cg*CKB2* for virulence. In the survival assay, immunocompromised ICR mice were injected with equal doses of *C. glabrata* and observed for up to 14 days. Mice infected with WT strains exhibited 50% mortality within 3 days, and only 25% percent of mice survived at the end of the experiment on day 14 (Fig. 6A). In contrast, Cg*CKB1* and Cg*CKB2* null mutants exhibited significant differences in survival curves with the wild-type strain (*p* < 0.05). Infection with Cg*CKB1* and Cg*CKB2* knock-out strains only caused 25% and 41.7% mortalities, respectively, and most mice recovered from infection (Fig. 6A).

Fungal burden was also measured 3 days post-infection in immunocompromised ICR mice. The mean number of wildtype yeast cells colonies (1.9 × 10^5^ CFUs/g) in the spleen (Fig. 6B) was significantly higher than in Cg*CKB1* null mutants (5.6 × 10^3^ CFUs/g, *p* < 0.001) and Cg*CKB2* null mutants (3.4 × 10^3^ CFUs/g, *p* < 0.001). Moreover, the fungal burden in the liver (Fig. 6C) was significantly higher in wildtype (9.9 × 10^4^ CFUs/g) than in Δ*ckb1* (1.1 × 10^4^ CFUs/g, *p* < 0.0001) and Δ*ckb2* (5.5 × 10^3^ CFUs/g, *p* < 0.0001). In addition, fungal burden in the kidney (Fig. 6D) was significantly higher in wildtype (3.1 × 10^4^ CFUs/g) than in Δ*ckb1* (2.3 × 10^3^ CFUs/g, *p* < 0.01) and Δ*ckb2* (1.6 × 10^3^ CFUs/g, *p* < 0.01).

Moreover, histological analysis confirmed these findings (Fig. 6E). Yeast colonies were observed in PAS (Periodic Acid-Schiff) stained kidney sections of WT-infected mice but were barely found in Δ*ckb1* and Δ*ckb2*-infected mice. In summary, the fungal burden is significantly decreased in Δ*ckb1* and Δ*ckb2* strains and Cg*CKB1* and Cg*CKB2* are required for organ colonization in the systemic candidiasis mouse model.

### Global transcriptional profiling analysis of Δ*ckb1* and Δ*ckb2* strains

**Fig. 7:**
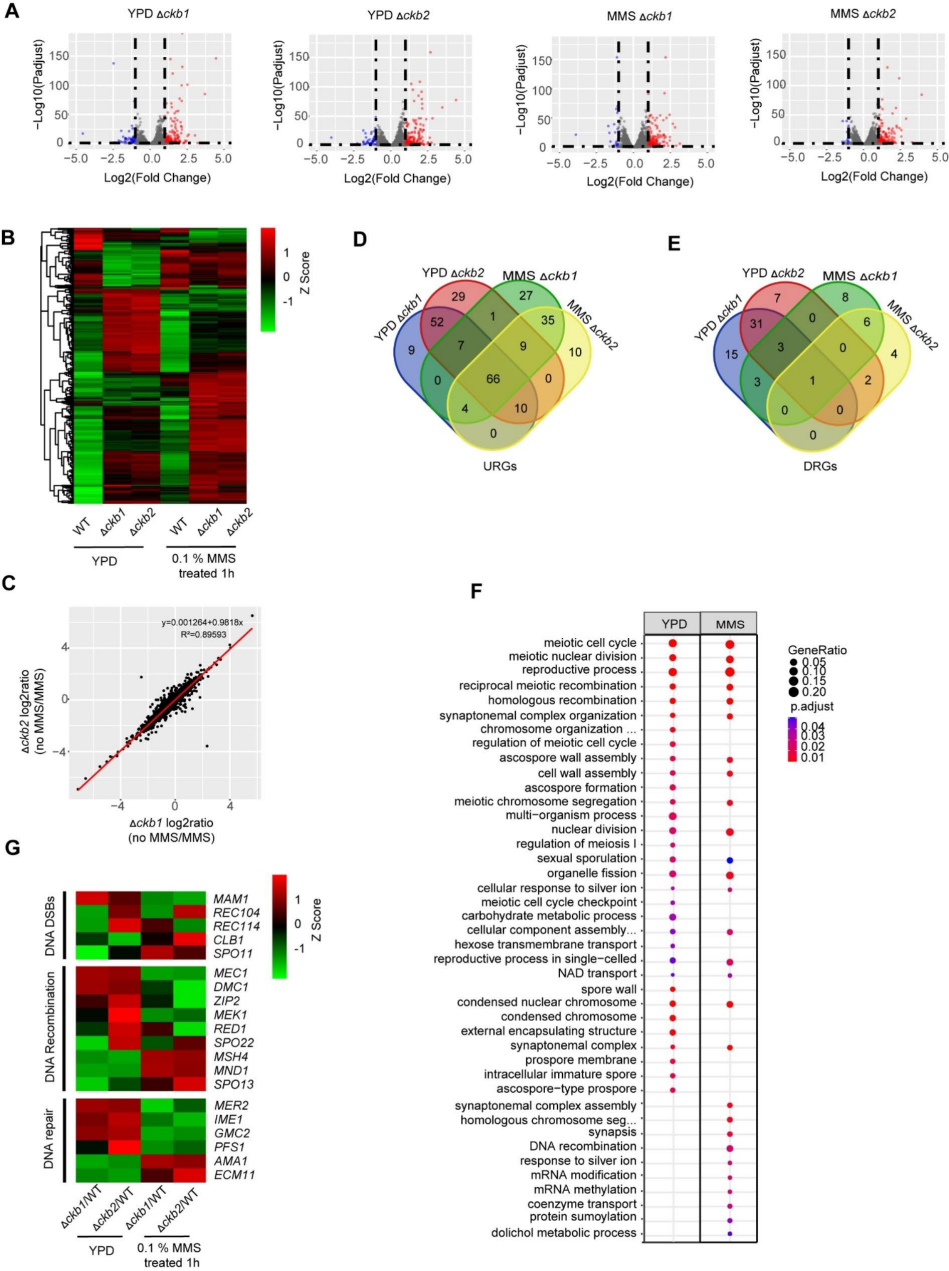
Transcriptional analysis in WT, Δ*ckb1*, and Δ*ckb2* strains. Log-phase yeast cells were inoculated into fresh YPD medium either with 0.01% MMS added or left untreated, total RNA was extracted and further sequenced. (**A**) Volcano plot of RNA seq transcriptome data displaying the gene expression pattern. The values of gene expression in each group were compared with those of WT strain under the same conditions. Significantly differentially expressed genes (FDR, *p* ≤ 0.05) are highlighted in red (upregulated) or blue (downregulated). (**B**) Heatmap plot of DEGs (Differential expression genes) displaying the pattern of gene expression. The gene expression value is normalized and converted to Z score. (**C**) Scatterplot where each gene is represented by a dot and its “no MMS/MMS” log2 ratio for *Δckb1* is plotted on the x axis and the ratio for *Δckb2* is plotted on the y axis. (**D** and **E**) Venn plot comparing the upregulated DEGs (URGs) and downregulated DEGs (DRGs) of *Δckb1* and *Δckb2* under all culture conditions. The differential genes of each group were compared with WT strain under the same conditions. (**F**) Gene ontology (GO) terms in the biological processes describing the upregulated and downregulated genes in each condition. The differential genes both in *Δckb1* and *Δckb2* (compared with WT under the same conditions) were clustered to GO analysis. (**G**) Heatmap of RNA-seq data for meiotic genes in Δ*ckb1* and Δ*ckb2* strains compared with WT under all culture conditions, including programmed formation of DNA double‐strand breaks (DSBs), recombination, and DNA repair coordination of the meiotic cell cycle.

To obtain a better understanding of molecular mechanisms underlying the DNA damage response in Δ*ckb1* and Δ*ckb2* mutants, transcriptome analyses were performed between WT, Δ*ckb1*, and Δ*ckb2* strains. Strains treated with 0.01% MMS for 1 h or untreated in YPD were analyzed (Fig. 7A). The transcriptome data revealed that Δ*ckb1* and Δ*ckb2* strains showed similar transcriptional profiles for YPD and YPD supplemented with 0.01% MMS (Fig. 7B C). The data also indicated that compared with WT, about 200 genes were up or downregulated in *Δckb1* and *Δckb2* strains with or without treatment with 0.01% MMS (Fig. 7D and E; Supplementary Table S2 and S3). This finding suggests a certain degree of overlap in the functions of ckb1 and ckb2 in *C. glabrata*.

To functionally characterize the transcriptional phenotypes, the differential genes found both in *Δckb1* and *Δckb2* (compared with WT under the same treatments) were analyzed by GO (Gene ontology) enrichment to identify key molecular processes. GO analysis highlighted that the differentially expressed genes in *Δckb1* and *Δckb2* were marked enriched in the processes related to the programmed formation of DNA double-strand breaks (DSBs), recombination, and DNA repair coordination of the meiotic cell cycle. In contrast to WT, the processes mentioned above were altered in *Δckb1* and *Δckb2* strains (Fig. 7F; Supplementary Table S4 and S5). It should be noted that after the deletion of *CgCKB1* and *CgCKB2*, the expression of many genes related to the meiotic cell cycle was upregulated, and these differences were magnified in the presence of 0.01% MMS (Fig. 7G). These genes included early meiotic genes *SPO11*, meiotic recombination-related gene *MSH4*, reciprocal meiotic recombination-related gene *REC104* and *REC114*, which have been associated with the repair of DSB of meiosis both in *S. cerevisiae* and *C. albicans* [23–26]. These findings suggested that *CgCKB1* and *CgCKB2* may maintain genome integrity, affecting the DNA damage response through certain pathways, such as cell cycle regulation.

### The role of CgCkb1 and CgCkb2 in *C. glabrata* intracellular survival

As the first defense line of the host against microbial invasion, macrophages recognize and engulf *C. glabrata* and then produce ROS (reactive oxygen species) or other active substances to induce DNA damage of *C. glabrata*. The role of CgCkb1 and CgCkb2 in intracellular survival of *C. glabrata* in macrophages and their involvement in DNA damage stress, led us to compare the genes regulated by CgCkb1 and CgCkb2 under control conditions or MMS treated with the activated genes of *C. glabrata* upon phagocytosis [10].

**Fig. 8:**
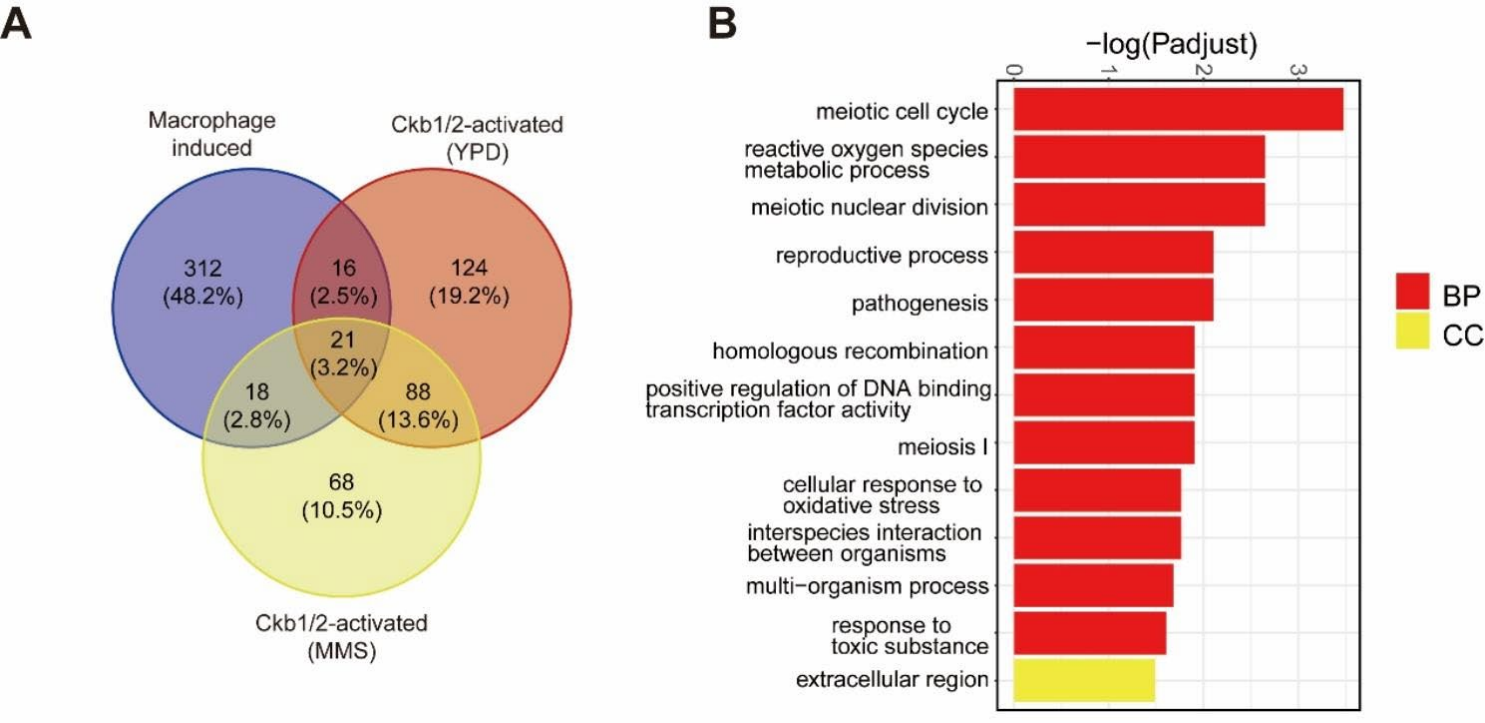
Transcriptome data reveal the link between Ckb1/2 and *C. glabrata* survival in macrophages. (**A**) Comparison of differential genes (compared with WT) regulated by *CgCKB1* and *CgCKB2* under control conditions and MMS conditions with the activated genes (compared with RPMI 1640 medium condition) of *C. glabrata* upon macrophage engulfment. (**B**) GO analysis demonstrates the biological processes involved in overlapping genes. A schematic diagram of a bar chart for the top enriched GO terms ranked according to the values of − log10 (adjusted p value). BP: Biological Process, CC: Cell Component.

A total of 55 genes overlapping were found, including *MSH4*, *IME1*, *MAM1*, *MEK1*, *RED1*, which involved in DNA DSB formation, DNA recombination and DNA repair processes (Fig. 8A; Supplementary Table S6 and S7). Based on the report of Rai.et al [10], 38 of 55 genes were down-regulated and 17 were up-regulated in macrophage-engulfed *C. glabrata* compared with control conditions. Interestingly, we noticed that a major part of these genes (34/38 and 9/17) exhibited the opposite expression patterns in Δ*Ckb1* and Δ*Ckb2*. These observations indicated the importance of CgCkb1 and CgCkb2 for *C. glabrata* in maintaining survival in macrophages. What is more, GO analysis indicated that these genes are involved in biological processes such as meiotic cell cycle, homologous recombination, and reactive oxygen species metabolism, which is consistent with our previous analysis (Fig. 8B; Supplementary Table S8). Therefore, these results suggesting that the response to DNA damage stress and the maintenance of genomic stability are crucial for intracellular survival of *C. glabrata.*

## Discussion

Antifungal resistance associated with *C. glabrata* has become a research hotspot in recent years; however, the pathogenicity of this life-threatening fungus remains unclear. Casein kinase 2 (CK2), a conserved ubiquitous serine/threonine kinase protein complex, has been extensively studied in many species and is advocated as a potential drug target for cancer. Nonetheless, no study has hitherto reported CK2 in *C. glabrata*. Herein, we identified roles for CgCkb1 and CgCkb2 as the regulatory subunits of CK2 and revealed their functions in response to DNA damage and maintaining a normal cell cycle. Moreover, we highlighted an association between genome integrity and virulence.

DNA damage is universal in life. DNA damage response is composed of DNA breaks repair and DNA damage checkpoint. In eukaryotic cells, broken DNA ends are detected and initiate DNA repair process. Meanwhile, a checkpoint is activated to delay cell cycle progression. In *S. cerevisiae*, receptors receive the signal of DNA damage and transfer it to central effectors through transduction factors [27]. The sensor kinase Mec1 recruits and phosphorylates the effector kinase Rad9 finally leading to the recruitment and phosphorylation of Rad53 [28]. The activated Rad53 can target multiple biological processes and is triggered upon DNA damage to promote cell cycle arrest and the transcription of DNA repair related genes [29]. Our RNA seq data indicated that the expression levels of *MEC1* in *Δckb1* and *Δckb2* significantly increased compared to WT either in YPD or under 0.01% MMS treatment. The evidence allows us to assume that Ckb1 and Ckb2 may be involved in the DNA damage response mediated by Mec1 in *C. glabrata*.

An increasing body of evidence suggests that cell cycle checkpoint and DNA repair pathways contribute to coping with DNA damage, and appropriate response to DNA damage is related to genome integrity and is vital for adaptation to surroundings [30, 31]. In *S. cerevisiae*, Spo11 forms a subcomplex with Ski8, Rec102, and Rec104, which promotes the formation of DSB together with the subcomplex formed by Rec114, Rec107, and Mei4, and then triggers the cells to enter the meiotic recombination process [24]. Studies in mammals, *S. cerevisiae*, and plants have found that Zip2 and Msh4 can promote the formation of synaptonemal complexes in meiosis [25]. Our study observed a delayed cell cycle in Cg*CKB1* or Cg*CKB2* null mutants. Meanwhile, the expression levels of *SPO11*, *REC102*, *REC104, REC114, MEI4*, *MSH4* and *ZIP2* were altered significantly in these strains. It has been established that these genes are functionally conserved and essential to cell cycle resumption and genomic stability. Accordingly, it is reasonable to infer that CgCkb1 and CgCkb2 might regulate the cell cycle through the above components. In the presence of DNA damage stress, eukaryotic cells trigger intricate DNA damage checkpoint programs to slow down the S-phase and prevent cell division [32, 33]. In *S. cerevisiae*, the cell cycle is governed by the expression level of transcription factor *PHO4* [34]. Our RNA seq data showed that the expression level of Cg*PHO4* does not decrease in *Δckb1* and *Δckb2* strains either in YPD or under DNA damage stress. Based on these findings, we hypothesize that the CK2 regulatory subunits do not regulate the cell cycle by inhibiting the transcription level of *PHO4* in *C. glabrata.* Nonetheless, more research is required to elucidate the specific mechanism of Ckb1/2 in cell cycle regulation in *C. glabrata.*

Although *C. albicans* has been studied comprehensively, there is still a knowledge gap on the virulence and infection processes of other non-*C. albicans* yeast, such as *C. glabrata.* In *C. albicans*, the catalytic subunit Cka2 of CK2 has been documented to play important roles in interactions with endothelial and oral epithelial cells in vitro and virulence in the murine model of oropharyngeal candidiasis [22]. There is no relevant report on the role of regulatory subunits (Ckb1/2) in virulence in *Candida* species currently. It is widely acknowledged that macrophages represent the first line of defense against infections. It has been stated that *C. glabrata* is a successful pathogen without promoting a robust immune response. It can replicate within macrophages to cause recalcitrant infections [7, 9]. Hence, protecting against DNA damage stress induced by macrophages might be essential for *C. glabrata* to adapt to the microenvironment. Rai et al. found that *C. glabrata* mutants defective in DNA repair (*Δrtt107*, Δ*sgs1*) exhibit attenuated virulence and *C. glabrata* remodeled its chromatin when interacting with macrophages, including chromatin structure modified, altered epigenetic signature, decreased protein acetylation, and so on [10]. In the present study, we examined the survival of CK2 mutant strains upon phagocytosis in THP-1 and RAW 264.7 cells. Contrary to WT, *Δckb1* and *Δckb2* exhibited significant deficiencies in replication inside macrophages, which indicated weakened adaption to the host and easier elimination of yeasts. The virulence of *Δckb1* and *Δckb2* was also attenuated in the candidiasis model. In addition, comparison the differential genes regulated by *CgCKB1* and *CgCKB2* with the activated genes of *C. glabrata* upon macrophage engulfment revealed that response to DNA damage is crucial for intracellular survival of *C. glabrata.* Overall, these observations provide compelling evidence that the response to DNA damage is essential for interaction with host immune and pathogenicity.

Some studies have reported the abnormal expression of CK2 in tumor cells, given that inhibitors of CK2 are widely thought of as potential drugs against cancer [35, 36]. There is evidence that CK2 inhibitors can effectively inhibit growth, hypha formation, and biofilm formation in *Candida* species [37, 38]. Based on the poor viability and attenuated virulence of *Δckb1* and *Δckb2*, inhibition of β subunits of CK2 in *C. glabrata* provides a novel approach for antifungal treatment.

## Conclusions

This is the first study to corroborate that CgCkb1 and CgCkb2 are required to respond to DNA damage and maintain a normal cell cycle. Moreover, CgCkb1 and CgCkb2 are crucial for the survival of engulfed *C. glabrata* and yield full virulence in the mouse model of invasive candidiasis. Our findings suggest that CgCkb1 and CgCkb2 might promote cellular recovery and adaptation of DSB and genomic stability, thereby maintaining the pathogenicity of *C. glabrata*. This study contributes to a better understanding of the interaction between *C. glabrata* and the host and paves the way for more efficient antifungal therapies.

## Materials and methods

### Strains and growth media

All strains used in this study were constructed from *C. glabrata* CBS138 and are listed in Table 1. Cg*CKB1*, Cg*CKB2*, Cg*CKA1* and Cg*CKA2* genes were disrupted using a recyclable, nourseothricin resistance marker through a homologous recombination strategy as described before [39]. The CEN/ARS episomal plasmid PCU-PDC1-GFP was used to construct Cg*CKB1*, Cg*CKB2*, Cg*CKA1* and Cg*CKA2* complemented strains. Briefly, genes were amplified form WT strains including the promoter and terminator regions and digested by SacI/XhoI. pCU-PDC1-GFP was digested by SacI/XhoI to remove GFP expression cassette. The residual plasmid backbone was ligated to digested gene fragments respectively to generate complemented plasmids. Plasmids were further transformed into *CgCKB1*, *CgCKB2*, Cg*CKA1* and Cg*CKA2* null mutants respectively using the lithium acetate method. Positive transformants grew on SD Medium-URA plates (synthetic medium containing 2% glucose and 2% agar without uracil, Solarbio, China) were picked and further verified. To exclude the potential effects of auxotrophic strain in cell and animal experiments, Ura + phenotype CBS138 was used as wildtype, and the mutant strains were generated from CBS138 through a homologous recombination strategy. Primers used are listed in supplementary Table S1.

Strains were cultured in YPD medium (Becton, Dickinson and Company, USA) composed of 1% yeast extract, 2% peptone, and 2% glucose, and gene-disrupted yeast transformants were selected on YPD plus 100 µg/mL nourseothricin (Sigma Aldrich, USA) as appropriate. Uracil auxotrophs were selected on YNB plates (Becton, Dickinson and Company, USA) composed of 0.67% yeast nitrogen base, 2% glucose, and 2% agar with amino acids and 0.1% 5-fluoroorotic acid (5-FOA, Sigma Aldrich, USA).

### Spot assays

Exponential phase WT, Δ*ckb1*, and Δ*ckb2* cells were washed with phosphate-buffered saline (PBS, Gibco, USA) and diluted to a series of concentrates from 10^7^ to 10^3^ CFU/mL [40]. 5µL dilutions were spotted on YPD plates containing DNA damage reagents methyl methanesulfonate (MMS, Sigma Aldrich, USA) or 4-Nitroquinoline-1-oxide (4-NQO, Sigma Aldrich, USA) then cultured at 30℃. Photos were taken 48 h later.

### Growth curve assay

Overnight inocula of WT, Δ*ckb1* and Δ*ckb2* in YPD medium were washed in PBS and diluted to OD_600_ of 0.02 in 200 µL YPD medium with 0.01% MMS or 4µM 4-NQO added, respectively. Dilutions were inoculated in a flat-bottomed 96-well plate, and the OD_600_ values were obtained by BioTek plate reader every 15 min at 30 °C [41]. Experiments were repeated at least three times and analyzed by GraphPad Prism Software.

**Table 1:** strains used in this study

Strain	Parent	Genotype	Description
CBS138	/	/	Wildtype strain; used for cell assays and animal models
WT	CBS138	CBS138/*ura3*△::*SAT1-FLIP*	Wildtype strain with *URA3* knock out; used for spot assays, cell apoptosis assays, TUNEL assays, cell cycle analysis and RNA-seq
△*ckb1*	WT	*ura3*△*ckb1*△::*SAT1-FLIP*	Used for spot assays, cell apoptosis assays, TUNEL assays, cell cycle analysis, and RNA-seq
△*ckb2*	WT	*ura3*△*ckb2*△::*SAT1-FLIP*	Used for spot assays, cell apoptosis assays, TUNEL assays, cell cycle analysis, and RNA-seq
△*cka1*	WT	*ura3*△*cka1*△::*SAT1-FLIP*	Used for spot assays
△*cka2*	WT	*ura3*△*cka2*△::*SAT1-FLIP*	Used for spot assays
△*ckb1::CKB1*	△*ckb1*	*ura3*△*ckb1*△::*SAT1-FLIP::pCU-CKB1*	Used for spot assays
△*ckb2*::CKB2	△*ckb2*	*ura3*△*ckb2*△::*SAT1-FLIP::pCU-CKB2*	Used for spot assays
△*cka1*::CKA1	△*cka1*	*ura3*△*cka1*△::*SAT1-FLIP::pCU-CKA1*	Used for spot assays
△*cka2*::CKA2	△*cka2*	*ura3*△*cka2*△::*SAT1-FLIP::pCU-CKA2*	Used for spot assays
△*ckb1*	CBS138	*ckb1*△::*SAT1-FLIP*	Used for cell assays and animal models
△*ckb2*	CBS138	*ckb2*△::*SAT1-FLIP*	Used for cell assays and animal models
△*epa1*	CBS138	*epa1*△::*SAT1-FLIP*	Used for cell assays

### Cell apoptosis assay and TUNEL assay

WT, Δ*ckb1* and Δ*ckb2* were cultured to exponential phase in RPMI 1640 medium and diluted to an OD_600_ of 0.2 and were either treated with 0.01% MMS or 4 µM 4-NQO for 2 h or left untreated. Then cells were collected and washed with PBS three times. Annexin V-FITC apoptosis detection kit (Beyotime Biotechnology, China) was used. Cells were double stained with Annexin V-FITC and PI (Propidium Iodide, Thermo Fisher Scientific, USA). Fluorescence intensity was detected by BD Fortessa Flow cytometer and data were analyzed by FlowJo software [42].Briefly, forward scatter area (FSC-A) vs. side scatter area (SSC-A) gate was used to gate out obvious debris. Then single cells were identified by side scatter area (SSC-A) vs. side scatter height (SSC-H) gate. The quadrant gates of Annexin V FITC-A vs. PI-A showed the non-apoptotic cells (Annexin V-FITC-/PI-), apoptotic cells (Annexin V-FITC+/PI-), necrotic cells (Annexin V-FITC+/PI+) and dead cells (Annexin V-FITC-/PI+), respectively.

The one-step TUNEL detection kit (Beyotime Biotechnology, China) was used for the TUNEL assay [43]. Briefly, log phase cells were treated with 0.01% MMS or 4 µM 4-NQO for 12 h at 30℃. Cells were digested with 10U Zymolyase 20T (Seikagaku Biobusiness, Japan) at 35℃ for 30 min and then fixed with 4% paraformaldehyde. Cells were incubated in a permeabilization solution for 5 min. For positive control, cells were treated with 2000 U DNase I for 30 min at 37℃. The cells were further incubated with a TUNEL reaction mixture and were observed under fluorescence microscopy or analyzed by flow cytometry. As for negative control, the TdT enzyme in TUNEL reaction mixture was replaced by ddH2O. The same strategies were applied to gate out obvious debris and identify single cells as described above. The proportions of TUNEL + cells were determined by the quantification of fluorescence intensity.

### Cell cycle analysis

2 × 10^8^ CFU/mL of WT, Δ*ckb1*, and Δ*ckb2* yeast cells were synchronized in YP (1% yeast extract and 2% peptone) for 24 h, respectively. Then yeast cells were released to YPD with or without 0.01% MMS at 30℃, 200 rpm, and collected once an hour. After being washed with PBS, aliquots were fixed with 70% pre-cold ethanol and stored at -20℃ overnight. Samples were further resuspended in 1 mL 0.2 M Tris-HCl, sonicated, and treated with RNase (Thermo Fisher Scientific, USA) at 37℃ overnight. After being washed with 0.2 M Tris-HCl, samples were stained with 0.05 mg/mL PI at 0℃ for 15 min, finally pelleted and resuspended in 0.2 M Tris-HCl with 0.01 mg/mL PI added. At least 50,000 events were analyzed by flow cytometry using BD Fortessa, and data were analyzed by FlowJo software [44]. The same strategies as in cell apoptosis assay were used to discard cell debris and identify the single cells. DNA content was measured by the PI histogram plot. The DNA content of G2 phase cells (2 C) is twice that of G1 phase cells (1 C). G1 and G2 phases cells were distinguished by different fluorescence intensity. G2 phase cells displayed approximately twice brighter fluorescence than G1 phase cells.

### Survival upon phagocytosis and adhesion assays

Human monocyte THP-1 cells (ATCC TIB-202) were cultured in RPMI1640 medium supplemented with 10% fetal bovine serum and 100 U/ml penicillin, and 100 µg/mL streptomycin (Thermo Fisher Scientific, USA ) at 37℃, 5% CO_2_. First, THP-1 cells were seeded into 24-well plates at a density of 5 × 10^5^ cells per well with 50 ng/mL of phorbol 12-myristate 13-acetate (PMA, Sigma Aldrich, USA) added. After 18 h, a fresh medium without PMA was replaced, and cells were cultured for another 18 h. Mouse leukemia macrophages RAW264.7 (ATCC TIB-71) were cultured in complete Dulbecco’s modified Eagle’s medium (DMEM) supplemented with 2 mM L-Glutamine, 100 U/ml penicillin, and 100 µg/mL streptomycin (Thermo Fisher Scientific, USA) at 37℃ under a 5% CO_2_ atmosphere. Afterward, *C. glabrata* cells were cocultured with THP-1 or RAW264.7 cells at MOI 1:10. 2 h later, supernatants were removed, and cells were washed with PBS three times. 300 µL 0.05% Triton X-100 (w/v) was added into per well to lyse macrophages, and the lysates were diluted and plated on YPD to calculate the number of yeast cells phagocyted by macrophages [45]. As for *C. glabrata* survival upon phagocytosis, THP-1 or RAW264.7 cells were cultured for 24 h after being washed with PBS and then lysed with the same method. Survival rates were calculated by the ratio of 24 h to 2 h yeast cell numbers. Experiments were performed three times.

As described before, adhesion assays were performed using human colorectal adenocarcinoma cells (Caco-2 ATCC HTB-37) and were repeated three times [39]. Δ*epa1* was verified as deficient in adherence and was used for control.

### Biofilm formation

5 × 10^7^ Log-phased yeast cells in YPD were collected and seeded in a 24-well plate, followed by a 37℃ incubation for 90 min. RPMI 1640 medium with 10% FBS contained was added to each well after being washed with PBS twice. Then the plate was incubated at 37℃ for 24 h and replaced with the fresh medium to incubate for another 24 h. Unattached yeast cells were washed with PBS three times. After 30 min of staining with crystal violet [0.4% (w/v)], 95% ethanol was used for destaining. The destaining solution was measured for absorbance at 595 nm. Wells without yeast cells were used as background [46]. Three biological replicates were performed.

### Animal candidiasis models

The virulence of WT, Δ*ckb1* and Δ*ckb2* strains were evaluated by mouse models of invasive candidiasis [47, 48]. Both survival curves of mouse and fungal burdens were assessed. Female outbred ICR mice (5–6 weeks old, 24–26 g) were obtained from Charles River Company, China. As for survival assays, 200 mg/kg cyclophosphamide (Sigma Aldrich, USA) was intraperitoneally injected per mouse on day − 3 and every fourth day later. Mice were randomly designated into groups according to the random number table method, with a total of 12 mice in each group. Then 1 × 10^8^ yeast cells in 200 µL in 0.9% (w/v) saline were injected through the tail veil on day 0. The survival conditions were monitored and recorded. Mice in the agonal stage were humanely euthanized by cervical dislocation. Experiments were terminated on day 14. As for fungal burden assays, groups of 6 mice were immunosuppressed with 200 mg/kg cyclophosphamide on day − 3 and were further injected with 5 × 10^7^ yeast cells through the tail veil on day 0. Mice were sacrificed on day 3 post-infection, and organs (spleen, liver, and kidney) were weighed and transferred to ice-cold PBS. Tissue homogenates were diluted, plated onto YPD agar, and incubated at 30℃ for 2 days. CFUs were calculated and analyzed. Evaluation of histology was performed by kidney tissue sections stained with hematoxylin-eosin (HE) and periodic acid-Schiff (PAS) (Servicebio Technology company, China).

### RNA sequencing

Log phase WT, Δ*ckb1*, and Δ*ckb2* cells were inoculated into fresh YPD medium either with 0.01% MMS added or left untreated. After 1 h of incubation, yeast cells were harvested, and total RNA was extracted by the acid phenol method [49]. Three biological replicates were performed per condition. RNA was frozen at -80℃ and sent to Shanghai Majorbio Bio-pharm Technology Co., Ltd (Shanghai, China) for further sequencing by Illumina novaseq6000 PE150. Quality-filtered RNA-seq reads were aligned to the *C. glabrata* genome (http://www.candidagenome.org) by STAR software (version 2.7.4). Gene expressions were obtained and normalized by StringTie (V2.1.2) and DESeq2 (V1.20). Significantly expressed genes were screened based on a 2-fold cut-off and *p*-value < 0.05.

### Electronic supplementary material

Below is the link to the electronic supplementary material.

Supplementary Material 1

Supplementary Material 2

Supplementary Material 3

## Data Availability

The dataset generated from this study has been deposited in the NCBI database under the GEO accession number GSE232439 (https://www.ncbi.nlm.nih.gov/geo/query/acc.cgi?acc=GSE232439). All primary data that support the findings of this study are available from the corresponding author upon reasonable request.

## Abbreviations

4-NQO: 4-Nitroquinoline-1-oxide
CFU: Colony-forming unit
DAPI: Diamidino-phenylindole
DEG: Differential expression gene
DMEM: Dulbecco’s modified Eagle’s medium
DSB: DNA double-strand break
GO: Gene ontology
HE: Hematoxylin-Eosin
MMS: Methyl methane sulfonate
OD: Optical density
PAS: Periodic acid-Schiff
PBS: Phosphate-buffered saline
PI: Propidium Iodide
PMA: Phorbol 12-myristate 13-acetate
PS: Phosphatidylserine
ROS: Reactive oxygen species
RNS: Reactive nitrogen species
SSB: Single-strand breaks
WT: Wild-type
YPD: Yeast extract peptone dextrose medium

## References

[1] Kumar K, Askari F, Sahu MS, Kaur R. *Candida Glabrata*: a Lot more than meets the Eye. Microorganisms. 2019;7(2). 10.3390/microorganisms7020039 PMC6407134 30704135

[2] Lamoth F, Lockhart SR, Berkow EL, Calandra T (2018). Changes in the epidemiological landscape of invasive candidiasis. J Antimicrob Chemother.

[3] Lotfali E, Fattahi A, Sayyahfar S, Ghasemi R, Rabiei MM, Fathi M (2021). A review on Molecular mechanisms of Antifungal Resistance in *Candida Glabrata*: Update and recent advances. Microb Drug Resist.

[4] Castanheira M, Deshpande LM, Davis AP, Carvalhaes CG, Pfaller MA (2022). Azole resistance in *Candida Glabrata* clinical isolates from global surveillance is associated with efflux overexpression. J Glob Antimicrob Resist.

[5] Timmermans B, De Las Penas A, Castano I, Van Dijck P. Adhesins in *Candida Glabrata*. J Fungi (Basel). 2018;4(2). 10.3390/jof4020060 PMC6023314 29783771

[6] Frias-De-Leon MG, Hernandez-Castro R, Conde-Cuevas E, Garcia-Coronel IH, Vazquez-Aceituno VA, Soriano-Ursua MA et al. *Candida Glabrata* Antifungal Resistance and virulence factors, a perfect pathogenic combination. Pharmaceutics. 2021;13(10). 10.3390/pharmaceutics13101529 PMC8538829 34683822

[7] Shantal CN, Juan CC, Lizbeth BS, Carlos HJ, Estela GB (2022). *Candida Glabrata* is a successful pathogen: an artist manipulating the immune response. Microbiol Res.

[8] Johnson CJ, Kernien JF, Hoyer AR, Nett JE (2017). Mechanisms involved in the triggering of neutrophil extracellular traps (NETs) by *Candida Glabrata* during planktonic and biofilm growth. Sci Rep.

[9] Kasper L, Seider K, Hube B (2015). Intracellular survival of *Candida Glabrata* in macrophages: immune evasion and persistence. FEMS Yeast Res.

[10] Rai MN, Balusu S, Gorityala N, Dandu L, Kaur R (2012). Functional genomic analysis of *Candida glabrata*-macrophage interaction: role of chromatin remodeling in virulence. PLoS Pathog.

[11] Steenwyk JL. Evolutionary divergence in DNA damage responses among Fungi. mBio. 2021;12(2). 10.1128/mBio.03348-20 PMC8092291 33727357

[12] Guillemain G, Ma E, Mauger S, Miron S, Thai R, Guerois R (2007). Mechanisms of checkpoint kinase Rad53 inactivation after a double-strand break in *Saccharomyces cerevisiae*. Mol Cell Biol.

[13] Roffey SE, Litchfield DW. CK2 regulation: perspectives in 2021. Biomedicines. 2021;9(10). 10.3390/biomedicines9101361 PMC8533506 34680478

[14] Bibby AC, Litchfield DW (2005). The multiple personalities of the regulatory subunit of protein kinase CK2: CK2 dependent and CK2 Independent roles reveal a secret identity for CK2beta. Int J Biol Sci.

[15] Loizou JI, El-Khamisy SF, Zlatanou A, Moore DJ, Chan DW, Qin J (2004). The protein kinase CK2 facilitates repair of chromosomal DNA single-strand breaks. Cell.

[16] Becherel OJ, Jakob B, Cherry AL, Gueven N, Fusser M, Kijas AW (2010). CK2 phosphorylation-dependent interaction between aprataxin and MDC1 in the DNA damage response. Nucleic Acids Res.

[17] Chen F, Huang X, Wu M, Gou S, Hu W (2017). A CK2-targeted pt(IV) prodrug to disrupt DNA damage response. Cancer Lett.

[18] Krebs JE (2007). Moving marks: dynamic histone modifications in yeast. Mol Biosyst.

[19] Talbert PB, Henikoff S (2017). Histone variants on the move: substrates for chromatin dynamics. Nat Rev Mol Cell Biol.

[20] Chantalat L, Leroy D, Filhol O, Nueda A, Benitez MJ, Chambaz EM (1999). Crystal structure of the human protein kinase CK2 regulatory subunit reveals its zinc finger-mediated dimerization. EMBO J.

[21] Canton DA, Zhang C, Litchfield DW (2001). Assembly of protein kinase CK2: investigation of complex formation between catalytic and regulatory subunits using a zinc-finger-deficient mutant of CK2beta. Biochem J.

[22] Chiang LY, Sheppard DC, Bruno VM, Mitchell AP, Edwards JE, Filler SG (2007). Candida albicans protein kinase CK2 governs virulence during oropharyngeal candidiasis. Cell Microbiol.

[23] Mu X, Murakami H, Mohibullah N, Keeney S (2020). Chromosome-autonomous feedback down-regulates meiotic DNA break competence upon synaptonemal complex formation. Genes Dev.

[24] Koehn DR, Haring SJ, Williams JM, Malone RE (2009). Tethering recombination initiation proteins in *Saccharomyces cerevisiae* promotes double strand break formation. Genetics.

[25] Zhang Q, Ji SY, Busayavalasa K, Yu C (2019). SPO16 binds SHOC1 to promote homologous recombination and crossing-over in meiotic prophase I. Sci Adv.

[26] Malkova A, Klein F, Leung WY, Haber JE (2000). HO endonuclease-induced recombination in yeast meiosis resembles Spo11-induced events. Proc Natl Acad Sci U S A.

[27] Elledge SJ (1996). Cell cycle checkpoints: preventing an identity crisis. Science.

[28] Waterman DP, Haber JE, Smolka MB (2020). Checkpoint responses to DNA double-strand breaks. Annu Rev Biochem.

[29] Pardo B, Crabbe L, Pasero P. Signaling pathways of replication stress in yeast. FEMS Yeast Res. 2017;17(2). 10.1093/femsyr/fow101 27915243

[30] Shor E, Perlin DS (2021). DNA damage response of major fungal pathogen *Candida Glabrata* offers clues to explain its genetic diversity. Curr Genet.

[31] Thomson GJ, Hernon C, Austriaco N, Shapiro RS, Belenky P, Bennett RJ (2019). Metabolism-induced oxidative stress and DNA damage selectively trigger genome instability in polyploid fungal cells. EMBO J.

[32] Branzei D, Foiani M (2010). Maintaining genome stability at the replication fork. Nat Rev Mol Cell Biol.

[33] Friedel AM, Pike BL, Gasser SM (2009). ATR/Mec1: coordinating fork stability and repair. Curr Opin Cell Biol.

[34] Ikeh M, Ahmed Y, Quinn J. Phosphate Acquisition and Virulence in Human Fungal pathogens. Microorganisms. 2017;5(3). 10.3390/microorganisms5030048 PMC5620639 28829379

[35] Spinello Z, Fregnani A, Quotti Tubi L, Trentin L, Piazza F, Manni S. Targeting protein kinases in Blood Cancer: focusing on CK1alpha and CK2. Int J Mol Sci. 2021;22(7). 10.3390/ijms22073716 PMC8038136 33918307

[36] Thus YJ, De Rooij MFM, Swier N, Beijersbergen RL, Guikema JEJ, Kersten MJ (2023). Inhibition of casein kinase 2 sensitizes mantle cell Lymphoma to venetoclax through MCL-1 downregulation. Haematologica.

[37] Maslyk M, Janeczko M, Demchuk OM, Boguszewska-Czubara A, Golczyk H, Sieroslawska A (2018). A representative of arylcyanomethylenequinone oximes effectively inhibits growth and formation of hyphae in *Candida albicans* and influences the activity of protein kinases in vitro. Saudi Pharm J.

[38] Janeczko M, Maslyk M, Kubinski K, Golczyk H (2017). Emodin, a natural inhibitor of protein kinase CK2, suppresses growth, hyphal development, and biofilm formation of *Candida albicans*. Yeast.

[39] Ni Q, Wang C, Tian Y, Dong D, Jiang C, Mao E (2018). CgPDR1 gain-of-function mutations lead to azole-resistance and increased adhesion in clinical *Candida glabrata* strains. Mycoses.

[40] Wang Y, Mao Y, Chen X, Huang X, Jiang Z, Yang K et al. Homeostatic control of an iron repressor in a GI tract resident. Elife. 2023;12. 10.7554/eLife.86075 PMC10259491 37227051

[41] Mei Y, Jiang T, Zou Y, Wang Y, Zhou J, Li J (2020). FDA approved Drug Library Screening identifies Robenidine as a repositionable antifungal. Front Microbiol.

[42] Phillips AJ, Sudbery I, Ramsdale M (2003). Apoptosis induced by environmental stresses and amphotericin B in *Candida albicans*. Proc Natl Acad Sci U S A.

[43] Camarillo-Marquez O, Cordova-Alcantara IM, Hernandez-Rodriguez CH, Garcia-Perez BE, Martinez-Rivera MA, Rodriguez-Tovar AV. Antagonistic Interaction of *Staphylococcus aureus* Toward *Candida glabrata* During in vitro Biofilm Formation Is Caused by an Apoptotic Mechanism. Front Microbiol. 2018; 9:2031. 10.3389/fmicb.2018.02031 PMC6125415 30214437

[44] Shor E, Garcia-Rubio R, DeGregorio L, Perlin DS. A noncanonical DNA damage checkpoint response in a Major Fungal Pathogen. mBio. 2020;11(6). 10.1128/mBio.03044-20 PMC7773997 33323516

[45] Rasheed M, Battu A, Kaur R (2018). Aspartyl proteases in *Candida Glabrata* are required for suppression of the host innate immune response. J Biol Chem.

[46] Kumar K, Moirangthem R, Kaur R (2020). Histone H4 dosage modulates DNA damage response in the pathogenic yeast *Candida Glabrata* via homologous recombination pathway. PLoS Genet.

[47] Chew SY, Ho KL, Cheah YK, Ng TS, Sandai D, Brown AJP (2019). Glyoxylate cycle gene ICL1 is essential for the metabolic flexibility and virulence of *Candida Glabrata*. Sci Rep.

[48] Calcagno AM, Bignell E, Warn P, Jones MD, Denning DW, Muhlschlegel FA (2003). *Candida Glabrata* STE12 is required for wild-type levels of virulence and nitrogen Starvation induced filamentation. Mol Microbiol.

[49] Chen C, Noble SM (2012). Post-transcriptional regulation of the Sef1 transcription factor controls the virulence of *Candida albicans* in its mammalian host. PLoS Pathog.

