# Supplementary material for: The regulatory subunits of CK2 complex mediate DNA damage response and virulence in Candida Glabrata
Source: BMC Microbiol. 2023 Oct 28;23:317. doi: 10.1186/s12866-023-03069-4 (PMC10612253; doi:10.1186/s12866-023-03069-4)
Supplement: Supplementary file 1 — Supplementary Material 1 [file 12866_2023_3069_MOESM1_ESM.docx]

TableS1 Primers used in this study.

|  | **Primer sequence(5’ 3’)** | **Description** |
| --- | --- | --- |
| URA3-up | F:gccatcgttcaagccttagc  R:actggccgtcgttttacaacatagccgagagtgccttgat | Used for disrupting *ura3* |
| URA3-nat | F:atcaaggcactctcggctatgttgtaaaacgacggccagt  R:cggttgtaagatgatgttgctaggaaacagctatgaccatg |  |
| URA3-down | F:catggtcatagctgtttcctagcaacatcatcttacaaccg  R:aagagtaagcaaactagcgc |  |
| CKB1-up | F:AGTTCCGTTATCTCTTTGTC  R:ACTGGCCGTCGTTTTACAACGTCGTGACTGAACACTAAGCCAATAGCACC | Used for disrupting *ckb1* |
| CKB1-nat | F:CAGTCACGACGTTGTAAAACGACGGCCAGT  R:AATTTCACACAGGAAACAGCTATGACCATG |  |
| CKB1-down | F:CATGGTCATAGCTGTTTCCTGTGTGAAATTACAGCAATTCTCGTCATAAC  R: CTCAGAGCAATCTTCTTTAC |  |
| CKB2-up | F:AAACGCGGTATCCAGTAACT  R:ACTGGCCGTCGTTTTACAACGTCGTGACTGGCTCTATCACGGATACAATC | Used for disrupting *ckb2* |
| CKB2-nat | F:CAGTCACGACGTTGTAAAACGACGGCCAGT  R:AATTTCACACAGGAAACAGCTATGACCATG |  |
| CKB2-down | F:CATGGTCATAGCTGTTTCCTGTGTGAAATTATGAAAACCCACACAGAGAC  R: ATTGTAGGTTCCTCAGTCTG |  |
| CKA1-up | F:ATCCATGTCATTGAAGCCAC  R:ACTGGCCGTCGTTTTACAACGCTGTAGCCCGAAGATATTC | Used for disrupting *cka1* |
| CKA1-nat | F:GAATATCTTCGGGCTACAGCGTTGTAAAACGACGGCCAGT  R:CGATGTTTCCTTAGTCGATCggaaacagctatgaccatg |  |
| CKA1-down | F:catggtcatagctgtttccGATCGACTAAGGAAACATCG  R: ACAGGGACAATATGAGCAGC |  |
| CKA2-up | F:AGCGAGTTAACATGCGGTAC  R:ACTGGCCGTCGTTTTACAACTAATAGTGCAGTTCCGCAGC | Used for disrupting *cka2* |
| CKA2-nat | F:GCTGCGGAACTGCACTATTAGTTGTAAAACGACGGCCAGT  R:GTTCTTAGGCGATTCATATGggaaacagctatgaccatg |  |
| CKA2-down | F:catggtcatagctgtttccCATATGAATCGCCTAAGAAC  R:GTTGCTCGAGGACTACATAC |  |
| CKB1-AB | F:GCGAGCTCAAGCTAGCGGTCTCGATGGC  R:ccgctcgagAGTCAAGTCGTTTGATATGG | Used for genes ectopic expression in pCU-PDC1-GFP plasmids |
| CKB2-AB | F:CGgaattcCACTCGAAATAATTGGAAGG  R:ccgctcgagTTTCCCTTTCGCTCTCTGAC |  |
| CKA1-AB | F:GCGAGCTCATCCATGTCATTGAAGCCAC R:ccgctcgagCTGATGTGCTATTTGTTACC |  |
| CKA2-AB | F:GCGAGCTCAGCGAGTTAACATGCGGTAC R:ccgctcgagCTTGTTCTTGGTCTGGTACC |  |
