# Supplementary material for: The regulatory subunits of CK2 complex mediate DNA damage response and virulence in Candida Glabrata
Source: BMC Microbiol. 2023 Oct 28;23:317. doi: 10.1186/s12866-023-03069-4 (PMC10612253; doi:10.1186/s12866-023-03069-4)
Supplement: Supplementary file 3 — Supplementary Material 3 [file 12866_2023_3069_MOESM3_ESM.docx]

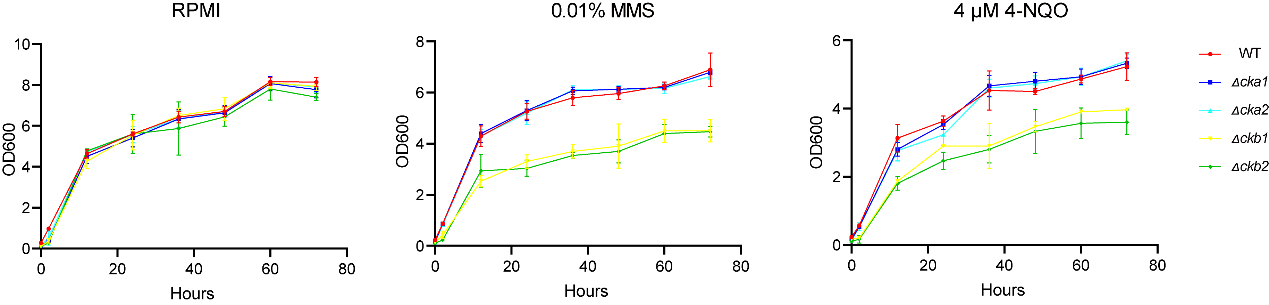


**Supplementary Figure 1**. Growth curve analysis of WT, Δ*ckb1* and Δ*ckb2* in RPMI 1640 medium. Yeast cells were inoculated in RPMI 1640 medium either in the absence or presence of 0.01% MMS or 4 μM 4-NQO. The optical density values were measured by Yoke Instrument UV721 spectrophotometer at 600 nm for 72 h. Experiments were repeated at least three times.


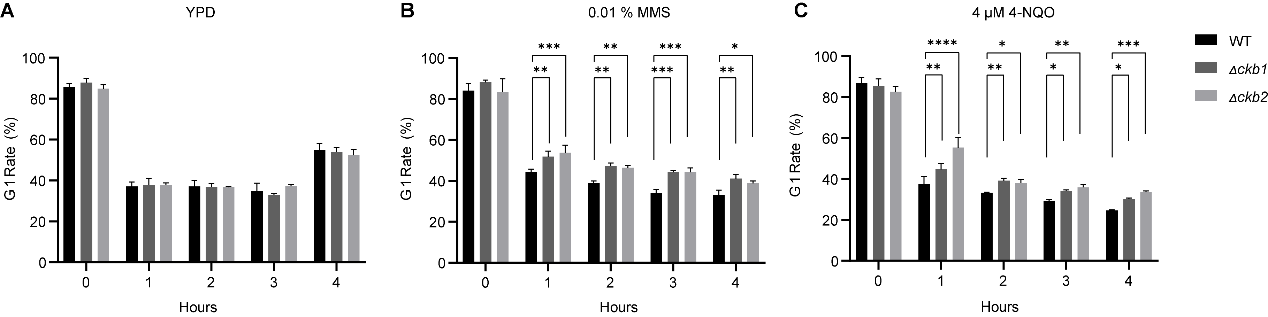


**Supplementary Figure 2.** The quantification of G1 phase cells in cell cycle analysis. The rates of G1 phase cells were determined by the fluorescence intensity of propidium iodide. Cells were cultured in YPD (A) or in the presence of 0.01 % MMS (B) or 4 μM 4-NQO (C) and harvested once an hour. The data were analyzed by one-way ANOVA followed by Tukey test; * p < 0.05,** p < 0.01, *** p < 0.001, **** p < 0.0001.
